# Dipeptidyl peptidase-4 inhibitors suppress myofibroblastic differentiation and hyaluronan synthesis in Graves’ orbitopathy fibroblasts

**DOI:** 10.3389/fendo.2026.1883214

**Published:** 2026-08-13

**Authors:** Chih-Wei Shih, Feng-Chiao Tsai, Shyang-Rong Shih, Shu-Lang Liao, I Chu, Tang-Meng Hong, Neng-Li Liao, Yi-Hsuan Wei

**Affiliations:** 1 Graduate Institute of Clinical Medicine, National Taiwan University College of Medicine, Taipei, Taiwan; 2 Department of Ophthalmology, Zhongxing Branch, Taipei City Hospital, Taipei, Taiwan; 3 Department of Ophthalmology, Good Will Clinic, Taipei, Taiwan; 4 Department of Internal Medicine, National Taiwan University Hospital, Taipei, Taiwan; 5 Department of Pharmacology, National Taiwan University College of Medicine, Taipei, Taiwan; 6 Department of Internal Medicine, National Taiwan University College of Medicine, Taipei, Taiwan; 7 Department of Ophthalmology, National Taiwan University Hospital, Taipei, Taiwan; 8 Department of Ophthalmology, National Taiwan University College of Medicine, Taipei, Taiwan

**Keywords:** dipeptidyl peptidase-4 inhibitors, fibrosis, Graves’ orbitopathy, hyaluronan, myofibroblastic differentiation, orbital fibroblasts, thyroid eye disease

## Abstract

Graves’ orbitopathy (GO) is characterized by inflammation, hyaluronan accumulation, tissue expansion, and fibrosis of orbital tissues, while effective antifibrotic medical therapies remain limited. Dipeptidyl peptidase-4 inhibitors (DPP4i), widely used for type 2 diabetes, have been reported to modulate fibroblast activity and fibrotic responses in several organs. This study investigated the effects of DPP4i on primary human orbital fibroblasts and explored their potential relevance to GO. Orbital fibroblasts were obtained from patients with GO and non-GO controls. DPP4 protein expression was assessed by Western blotting. The effects of linagliptin and sitagliptin on fibroblast proliferation, myofibroblastic differentiation, adipogenesis, and hyaluronan production were evaluated using DAPI nuclei counts, α-smooth muscle actin (α-SMA) immunofluorescence and Western blotting, Oil Red O staining, and ELISA, respectively. DPP4 protein was detected in orbital fibroblasts from both GO and non-GO groups. Linagliptin and sitagliptin significantly reduced transforming growth factor-β (TGF-β)-induced α-SMA expression in GO fibroblasts, whereas their effects in non-GO fibroblasts were modest. Both linagliptin and sitagliptin significantly suppressed interleukin-1β (IL-1β)-stimulated hyaluronan production in GO and non-GO fibroblasts. Neither inhibitor significantly altered DAPI-based cell number measurements or lipid accumulation in either group. These findings provide *in vitro* evidence that DPP4i modulate selected orbital fibroblast phenotypes related to myofibroblastic differentiation and cytokine-induced hyaluronan production. Further mechanistic, pharmacokinetic, *in vivo*, and clinical studies are warranted to clarify their relevance to GO-related tissue remodeling.

## 1 Introduction

Graves’ orbitopathy (GO), also known as thyroid eye disease or thyroid orbitopathy, affects 40% of patients with Graves’ disease (GD), as well as patients with hypothyroid autoimmune thyroiditis or without overt thyroid dysfunction (1, 2). Its pathogenesis involves autoimmune targeting the connective tissue around extraocular muscles and orbital fat. Orbital tissues in patients with GO are infiltrated by T cells, monocytes, macrophages, and B cells (3). These immune cells interact with autoantibodies against the thyroid-stimulating hormone receptor, which then activate IGF-1R through receptor crosstalk, and inflammatory cytokines, including interleukins, TNF-α, TGF-β, and interferon-γ, exacerbating inflammation and fibrosis (4–6). Orbital fibroblasts play a central role by secreting cytokines, producing excess hyaluronan, undergoing adipogenesis, and differentiating into myofibroblasts. These processes drive edema, tissue expansion, and fibrosis, making fibroblasts key therapeutic targets in GO (4, 7).

Clinical manifestations of GO range from periocular redness and swelling to proptosis, eyelid retraction, extraocular muscle dysfunction, and, in severe cases, vision-threatening compressive optic neuropathy. GO is traditionally described as a biphasic clinical course, beginning with an active inflammatory stage characterized by orbital tissue edema, expansion, and adipogenesis, and progressing into an inactive stage dominated by orbital tissue remodeling and fibrosis (8). However, emerging evidence suggests that fibrosis does not appear to be restricted to a late inactive phase, but may instead occur early in the disease course as part of the inflammatory process, possibly reflecting a tissue repair response (9). Once fibrosis is established within extraocular muscles and orbital connective tissue, functional recovery and therapeutic responsiveness are limited. Current treatments largely target inflammation during the active phase, whereas effective strategies to prevent or reverse orbital fibrosis remain limited (7, 10, 11). Intravenous glucocorticoids remain the first-line therapy for active, moderate-to-severe disease, while teprotumumab and tocilizumab are emerging biologic agents for treatment (7, 10–14). However, the clinical utility of these agents is constrained by adverse effects, including glucocorticoid-induced hepatotoxicity, teprotumumab-associated hearing loss or hyperglycemia, and increased infection risk with tocilizumab (10, 15, 16). Surgical intervention is often required for patients with poor response to medical therapy, those with vision-threatening disease, or those in the inactive phase with complications such as exophthalmos, muscle dysfunction, or eyelid malposition. These limitations highlight the unmet need for therapies that target the underlying mechanisms of orbital tissue remodeling and fibrosis in GO.

Dipeptidyl peptidase-4 (DPP4) is a membrane glycoprotein with protease activity, expressed on various cells, including epithelial cells, lymphocytes, and fibroblasts. It is also present in soluble form in serum (17). Beyond glucose metabolism, DPP4 regulates inflammatory and immune responses and contributes to autoimmune disease pathogenesis (18). Altered serum DPP4 levels have been reported in GD and GO, implicating it in disease development (19, 20). Moreover, DPP4 promotes fibrosis through inflammatory and fibroblast activation in organs such as the kidney, liver, heart, and skin (21–24).

DPP4 inhibitors (DPP4i), approved for type 2 diabetes since 2006, are well tolerated and carry a low risk of hypoglycemia. Beyond glucose lowering, DPP4i exert anti-inflammatory, anti-apoptotic, and immunomodulatory effects, with evidence suggesting their potential to prevent or reverse fibrosis in multiple tissues (21–26). Given that GO involves inflammation-driven fibrosis, we hypothesize that the anti-inflammatory and antifibrotic properties of DPP4i may benefit GO patients. This study aimed to evaluate the effects of DPP4i on orbital fibroblast behavior and explore their potential relevance as antifibrotic modulators in GO.

## 2 Methods

### 2.1 Cell culture

Patients undergoing orbital decompression surgery for GO and healthy subjects without GO undergoing cosmetic eyelid surgery at the National Taiwan University Hospital were recruited. Orbital tissues were collected after obtaining informed consent. Orbital fibroblasts were derived from these tissues. In total, ten patients with GO (GO group) and ten healthy subjects without GO (non-GO group) were included in the study. The characteristics of the study participants are summarized in **Table 1**. All patients in the GO group were euthyroid with a relatively low clinical activity score ranging from 1 to 3. Among patients with GO, two had received glucocorticoid therapy within 3 months before decompression surgery, and none had received other immunomodulators. Briefly, orbital tissues were minced in phosphate-buffered saline (PBS) and incubated with a sterile solution containing collagenase (125 U/mL; Thermo Fisher Scientific, Waltham, MA, USA) at 37 °C in a humidified incubator with 5% CO_2_. The digested tissues were centrifuged to pellet the cells, which were then resuspended in Dulbecco’s Modified Eagle’s Medium (DMEM/F-12; Gibco, Thermo Fisher Scientific, Waltham, MA, USA) supplemented with 20% fetal bovine serum (FBS) and 1% penicillin/streptomycin (P/S). Orbital fibroblasts were cultured and used between the second and fifth passages. Within each experiment, treatment conditions for the same donor were performed using cells at the same or adjacent passage to minimize passage-related variability. This study adhered to the ethical standards outlined in the Declaration of Helsinki and was approved by the Institutional Review Board of the National Taiwan University Hospital (protocol number: 201412026RINB).

**Table 1:** Characteristics of study subjects.

Variable	GO	Non-GO
Age (years)*	50 (37–52)	56 (48–67)
Sex (male: female)	3:7	5:5
fT4 (ng/dL)	1.2 (0.91–1.27)	—
TSH (mU/L)	0.500 (0.127–1.948)	—
TBII (%)	23.15 (6.6–50.5)	—
Clinical activity score	2 (1–3)	—

fT4, free thyroxine; GO, Graves’ orbitopathy; Non-GO, non-Graves’ orbitopathy; TBII, thyrotropin-binding inhibitory immunoglobulin; TSH, thyroid-stimulating hormone. Data are presented as the median (interquartile range). Comparisons between GO and non-GO groups were performed using the Wilcoxon–Mann–Whitney test for variables available in both groups. ^*^p < 0.05.

### 2.2 Proliferation assay

Orbital fibroblasts were seeded at 5,000 cells/well in collagen-coated 96-well plates and allowed to attach overnight. The medium was replaced with DMEM/F-12 containing 0.1% FBS and 1% P/S, with or without DPP4i (linagliptin [1µM] or sitagliptin [20µM]; Cayman Chemical, Ann Arbor, MI, USA) for 48 hours. The concentrations of linagliptin (1 μM) and sitagliptin (20 μM) were selected based on pilot experiments showing consistent inhibition of DPP4 enzymatic activity without apparent changes in cell morphology or cell number, as well as previous *in vitro* studies examining the effects of DPP4i on fibroblast activation and fibrosis-related responses (26–28). Fibroblast nuclei were stained with 4',6-diamidino-2-phenylindole (DAPI). DAPI-positive nuclei were counted in nine randomly selected non-overlapping fields per well at 20× magnification under a fluorescence microscope (Eclipse Ti; Nikon, Tokyo, Japan). Experiments were performed in technical triplicates for each donor and treatment condition.

### 2.3 Myofibroblastic differentiation assay

Fibroblasts were plated at 3,000 cells/well and incubated overnight. The medium was changed to DMEM/F-12 containing 0.1% FBS, 1% P/S, and TGF-β (10 ng/mL) for 48 hours, with or without DPP4i (linagliptin [1µM] or sitagliptin [20µM]). Cells were fixed with acetone/methanol (1:1) on ice for 5 min and blocked with 5% bovine serum albumin (BSA) in PBS for 1 hour. To detect α-smooth muscle actin (α-SMA), a marker of myofibroblastic differentiation, cells were incubated overnight at 4 °C with α-SMA primary antibodies (1:100 dilution; ab5694, Abcam, Cambridge, UK; RRID: AB_2223021) in PBS containing 1% BSA. Following this, cells were treated with Alexa Fluor 594 dye-conjugated secondary antibodies (1:300 dilution; anti-rabbit, A-11012, Invitrogen, Thermo Fisher Scientific, Waltham, MA, USA; RRID: AB_2534079) in PBS containing 1% BSA for 1 hour. Nuclei were stained using 10 μg/mL of DAPI in PBS for 15 minutes. Fluorescence signals for α-SMA and DAPI-stained nuclei were observed under a fluorescence microscope (Eclipse Ti; Nikon, Tokyo, Japan). The fluorescence intensity ratio of α-SMA to DAPI-stained nuclei was calculated to quantitatively evaluate myofibroblastic differentiation.

### 2.4 Adipogenesis assay

Fibroblasts were seeded at 5,000 cells/well in collagen-coated 96-well plates and incubated for 24 h. Subsequently, the cells were cultured in DMEM/F-12 supplemented with 20% FBS and 1% P/S, with or without DPP4i (linagliptin [1µM] or sitagliptin [20µM]) for 12 days. The culture medium was replaced every 2 days. After the incubation, cells were fixed with 4% paraformaldehyde and stained with Oil Red for lipid droplets and DAPI for nuclei. A fluorescence microscope (Eclipse Ti; Nikon, Tokyo, Japan) was used to assess the fluorescence of the oil droplets and nuclei. The fluorescence intensity ratio of Oil Red O to DAPI-stained nuclei was calculated to quantitatively evaluate adipogenesis of fibroblasts.

### 2.5 Hyaluronan ELISA assay

Orbital fibroblasts were seeded at 5,000 cells/well in collagen-coated 96-well plates and allowed to attach overnight. The culture medium was then replaced with DMEM/F-12 containing 0.1% FBS, 1% P/S, and IL-1β (10 ng/mL), with or without DPP4i (linagliptin [1 µM] or sitagliptin [20 µM]). After 24 hours, the culture supernatant was collected, and hyaluronan levels were measured using a specific enzyme-linked immunosorbent assay (ELISA) kit (Quantikine Hyaluronan ELISA, R&D Systems, Minneapolis, MN, USA; RRID: AB_2895403), following the manufacturer’s instructions.

### 2.6 Western blot analysis

Proteins were isolated from cultured fibroblast lysates. Equal amounts of protein (15 μg) were denatured in sample buffer, resolved on 10% SDS–PAGE, and transferred onto nitrocellulose membranes. After blocking with 4% BSA for 1 hour at room temperature, membranes were incubated overnight at 4 °C with primary antibodies, including DPP-4 (1:1000; #67138, Cell Signaling Technology, Danvers, MA, USA; RRID: AB_2728750), α-SMA (1:2000; ab5694, Abcam, Cambridge, UK; RRID: AB_2223021), phosphorylated Smad3 (p-Smad3; 1:3000; ab52903, Abcam, Cambridge, UK; RRID: AB_882596), and total Smad3 (1:3000; ab40854, Abcam, Cambridge, UK; RRID: AB_777979). Bound antibodies were detected with HRP-conjugated secondary antibody (anti-rabbit, #7074, Cell Signaling Technology; RRID: AB_2099233) and visualized using an ECL system (MilliporeSigma, Burlington, MA, USA) followed by X-ray film exposure. Band intensities were quantified using ImageJ. DPP4 and α-SMA signals were normalized to GAPDH as loading control. For Smad signaling analysis, phospho-Smad signals were normalized to total Smad. For donor-matched comparisons, normalized values were further expressed relative to the corresponding control from the same donor, which was set to 1.

### 2.7 Statistics

Fibroblast proliferation, α-SMA immunofluorescence, and Oil Red O–assessed adipogenesis assays were performed in technical triplicate for each donor and treatment condition. For quantitative analyses, values from technical replicates were averaged for each donor and normalized to the corresponding control from the same donor, which was set to 1, and used for statistical analysis. Western blot densitometric values were normalized within each donor, expressed as relative fold changes, and log-transformed before paired statistical analysis. Comparisons between treatment conditions were conducted using paired t-test for donor-matched samples. A two-sided P value < 0.05 was considered statistically significant. Statistical analyses were performed using Stata/SE 14.0 software for Windows (StataCorp LP, College Station, TX, USA).

## 3 Results

### 3.1 DPP4 expression in orbital fibroblasts

**Figure 1:**
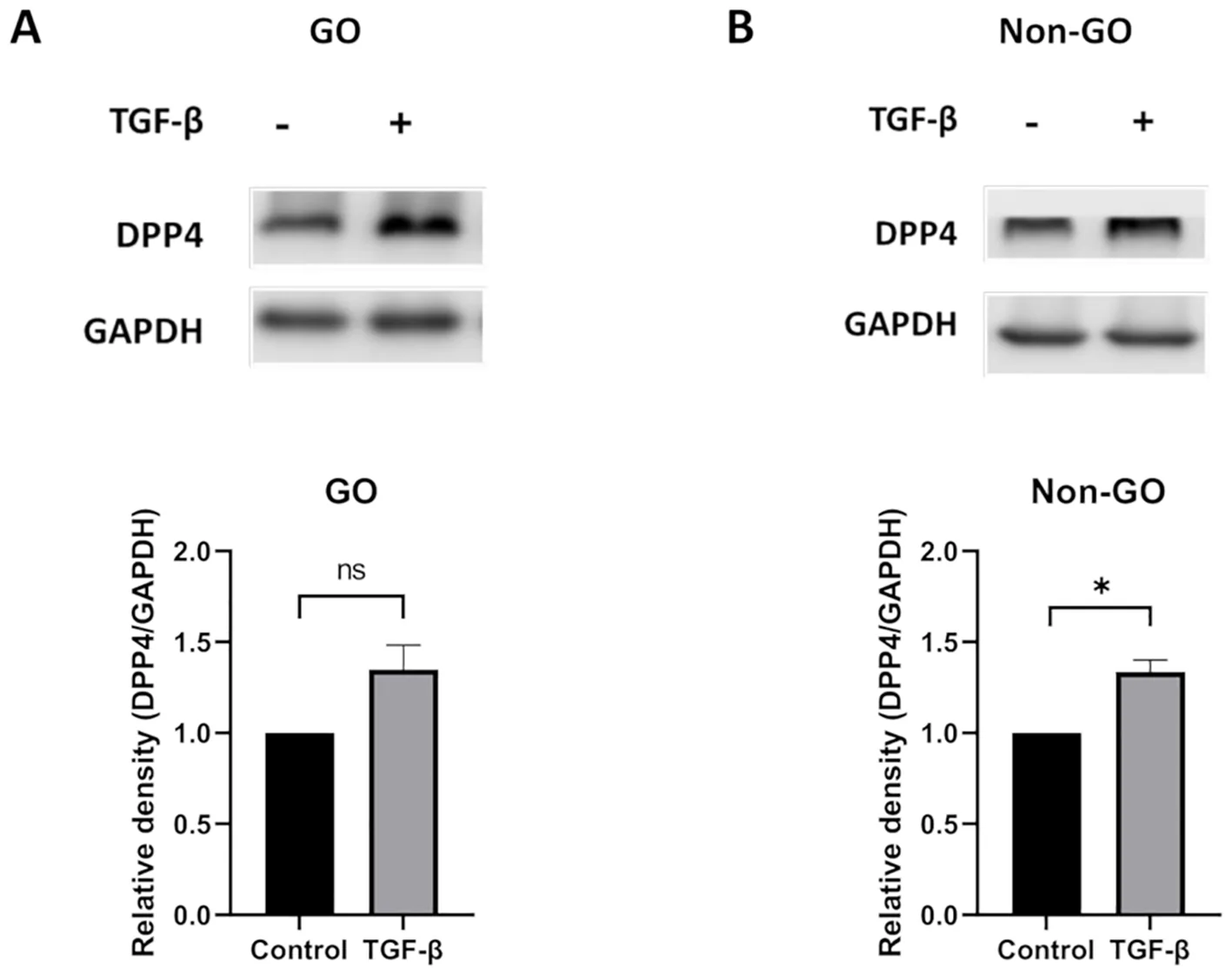
DPP4 protein expression in orbital fibroblasts from GO and non-GO subjects. Orbital fibroblasts were treated with or without transforming growth factor-β (TGF-β, 10 ng/mL) for 48 hours. **(A)** Representative Western blots and densitometric analysis of DPP4 protein expression in orbital fibroblasts derived from patients with GO (n = 5 donors). **(B)** Representative Western blots and densitometric analysis of DPP4 protein expression in orbital fibroblasts derived from non-GO controls (n = 5 donors). GAPDH was used as the loading control. Band intensities were quantified using ImageJ. DPP4 signals were normalized to GAPDH and further expressed relative to the corresponding untreated control from the same donor, which was set to 1. Data are presented as mean ± SD from biological donors. ns, not significant; *P < 0.05.

DPP4 protein was detected by Western blot in orbital fibroblasts from both GO and non-GO donors. DPP4 bands were observed in all samples, with or without TGF-β treatment for 48 hours. In GO fibroblasts, TGF-β stimulation produced a non-significant trend toward increased DPP4 expression. In donor-matched analysis, non-GO fibroblasts showed a significant upregulation of DPP4 following TGF-β treatment (**Figure 1**).

### 3.2 DPP4i did not affect orbital fibroblast proliferation

**Figure 2:**
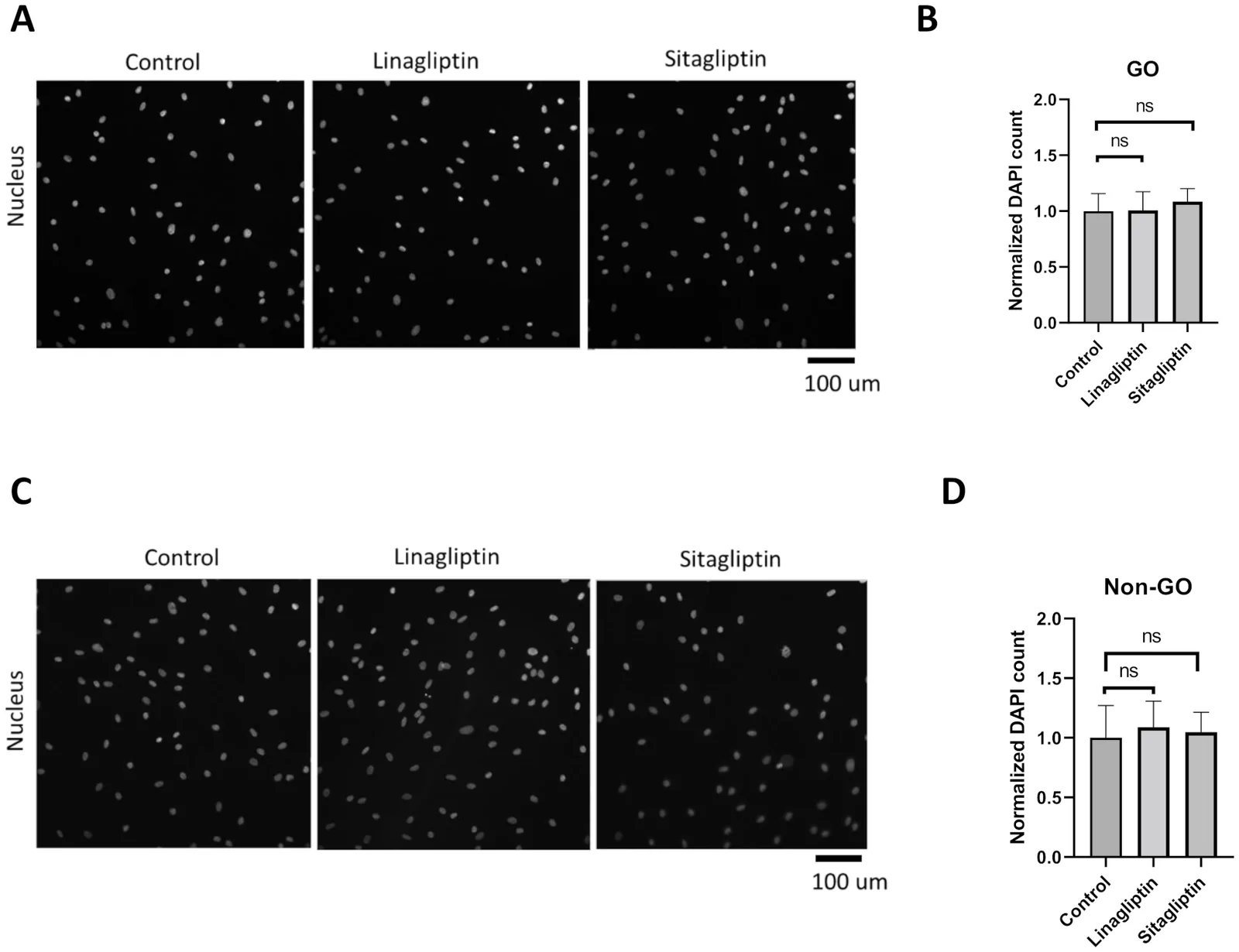
DPP4 inhibitors do not affect proliferation of orbital fibroblasts from GO or non-GO subjects. Orbital fibroblasts derived from GO (n = 10 donors) and non-GO (n = 10 donors) subjects were cultured in the presence or absence of DPP4 inhibitors, linagliptin (1 µM) or sitagliptin (20 µM), for 48 hours. Nuclei were stained with 4',6-diamidino-2-phenylindole (DAPI) to quantify cell numbers. **(A)** Representative DAPI staining images of GO orbital fibroblasts. **(B)** Quantification of DAPI-positive nuclei in GO orbital fibroblasts. **(C)** Representative DAPI staining images of non-GO orbital fibroblasts. **(D)** Quantification of DAPI-positive nuclei in non-GO orbital fibroblasts. DAPI-positive nuclei were counted in nine randomly selected non-overlapping fields per well at 20× magnification. Experiments were performed in technical triplicates for each donor and treatment condition. For each donor, technical replicates were averaged, and values were normalized to the corresponding untreated control, which was set to 1. Data are presented as mean ± SD from biological donors. ns, not significant.

Orbital fibroblast proliferation was assessed by DAPI staining to quantify nuclei. No significant differences in DAPI counts were observed after treatment with linagliptin (1 µM) or sitagliptin (20 µM) in either the GO group (**Figures 2A, B**) or the non-GO group (**Figures 2C, D**).

### 3.3 DPP4i significantly reduced myofibroblastic differentiation in the GO group

**Figure 3:**
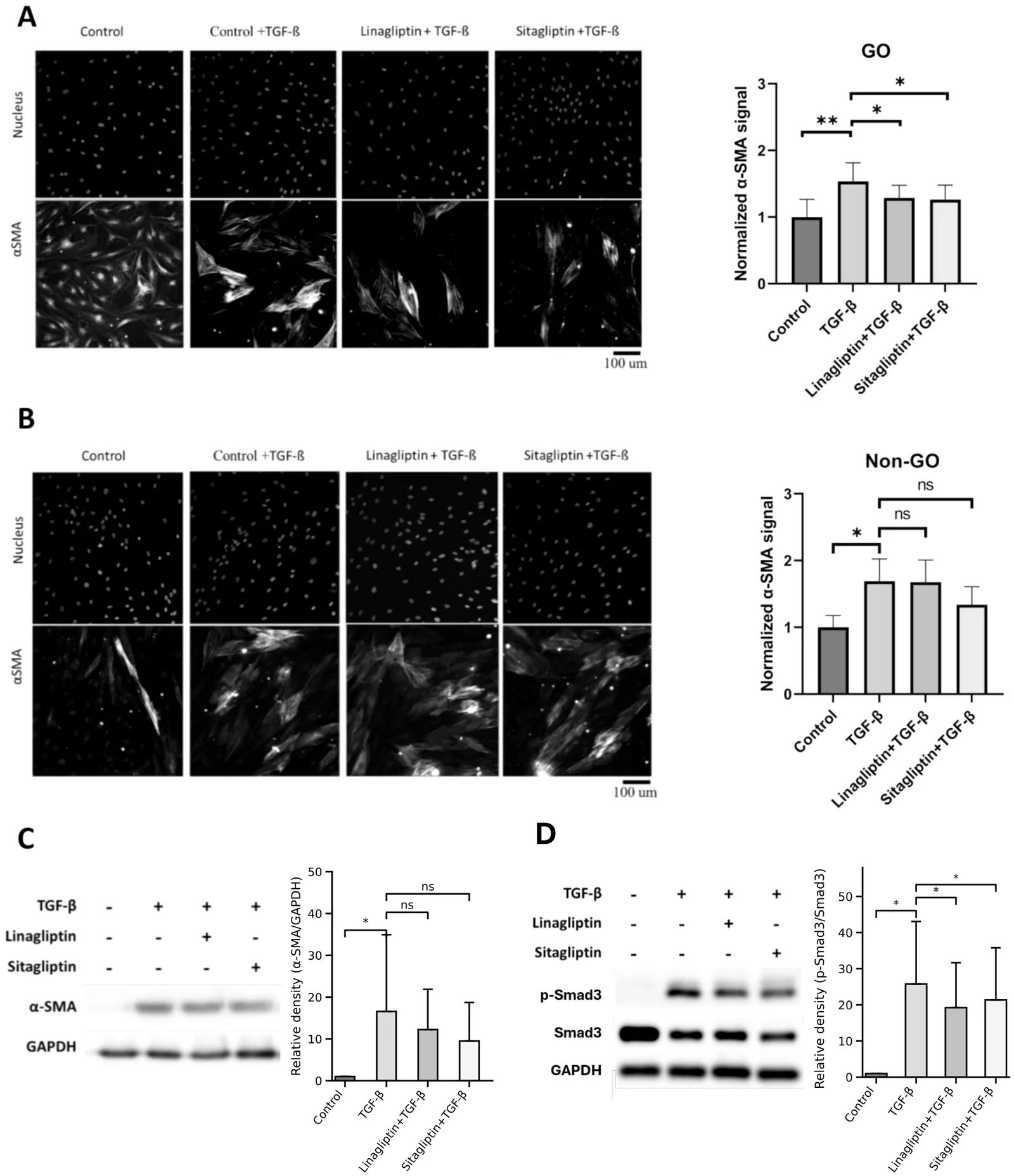
DPP4 inhibitors suppress TGF-β–induced myofibroblastic differentiation in GO orbital fibroblasts. Orbital fibroblasts derived from GO (n = 10 donors) and non-GO (n = 10 donors) subjects were stimulated with transforming growth factor-β (TGF-β, 10 ng/mL) in the presence or absence of DPP4 inhibitors, linagliptin (1 µM) or sitagliptin (20 µM), for 48 hours. **(A)** Representative immunofluorescence staining of α-smooth muscle actin (α-SMA) and quantification of normalized α-SMA fluorescence intensity in GO orbital fibroblasts. **(B)** Representative immunofluorescence staining of α-SMA and quantification of normalized α-SMA fluorescence intensity in non-GO orbital fibroblasts. α-SMA fluorescence intensity was normalized to DAPI-stained nuclei. For each donor, technical replicates were averaged, and values were normalized to the corresponding untreated control, which was set to 1. **(C)** Western blot analysis of α-SMA expression in GO orbital fibroblasts (n = 5 donors), performed as supportive protein-level validation, with GAPDH used as the loading control. α-SMA band intensities were quantified using ImageJ, normalized to GAPDH, and further expressed relative to the corresponding untreated control from the same donor, which was set to 1. **(D)** Western blot analysis of phosphorylated Smad3 (p-Smad3) and total Smad3 in GO orbital fibroblast lysates (n = 3 donors). p-Smad3/Smad3 were further normalized to the corresponding untreated control from the same donor, which was set to 1. Data are presented as mean ± SD from biological donors. ns, not significant; *P < 0.05; **P < 0.01.

α-SMA staining showed that TGF-β stimulation significantly increased α-SMA expression in both GO and non-GO groups (ten donors in each group). Linagliptin (1 µM) and sitagliptin (20 µM) significantly reduced TGF-β–induced myofibroblastic differentiation in GO fibroblasts, whereas in non-GO fibroblasts sitagliptin (20 µM) showed only a non-significant trend toward reduction (**Figures 3A, B**). Western blot analysis of α-SMA was performed using GO fibroblast lysates from five biological donors as supportive protein-level validation. TGF-β increased α-SMA protein expression, and sitagliptin showed a consistent decreasing trend across all five donors, whereas linagliptin showed a weaker and more variable effect. However, these changes did not reach statistical significance (**Figure 3C**). To explore whether DPP4i affected TGF-β/Smad signaling, p-Smad3 and total Smad3 were examined by Western blotting in a subset of GO orbital fibroblast lysates in three donors. In this exploratory subset, TGF-β significantly increased the p-Smad3/Smad3 ratio. Both linagliptin and sitagliptin significantly reduced TGF-β–induced p-Smad3/Smad3 ratio (**Figure 3D**).

### 3.4 DPP4i did not affect Oil Red O–assessed adipogenesis in orbital fibroblasts

**Figure 4:**
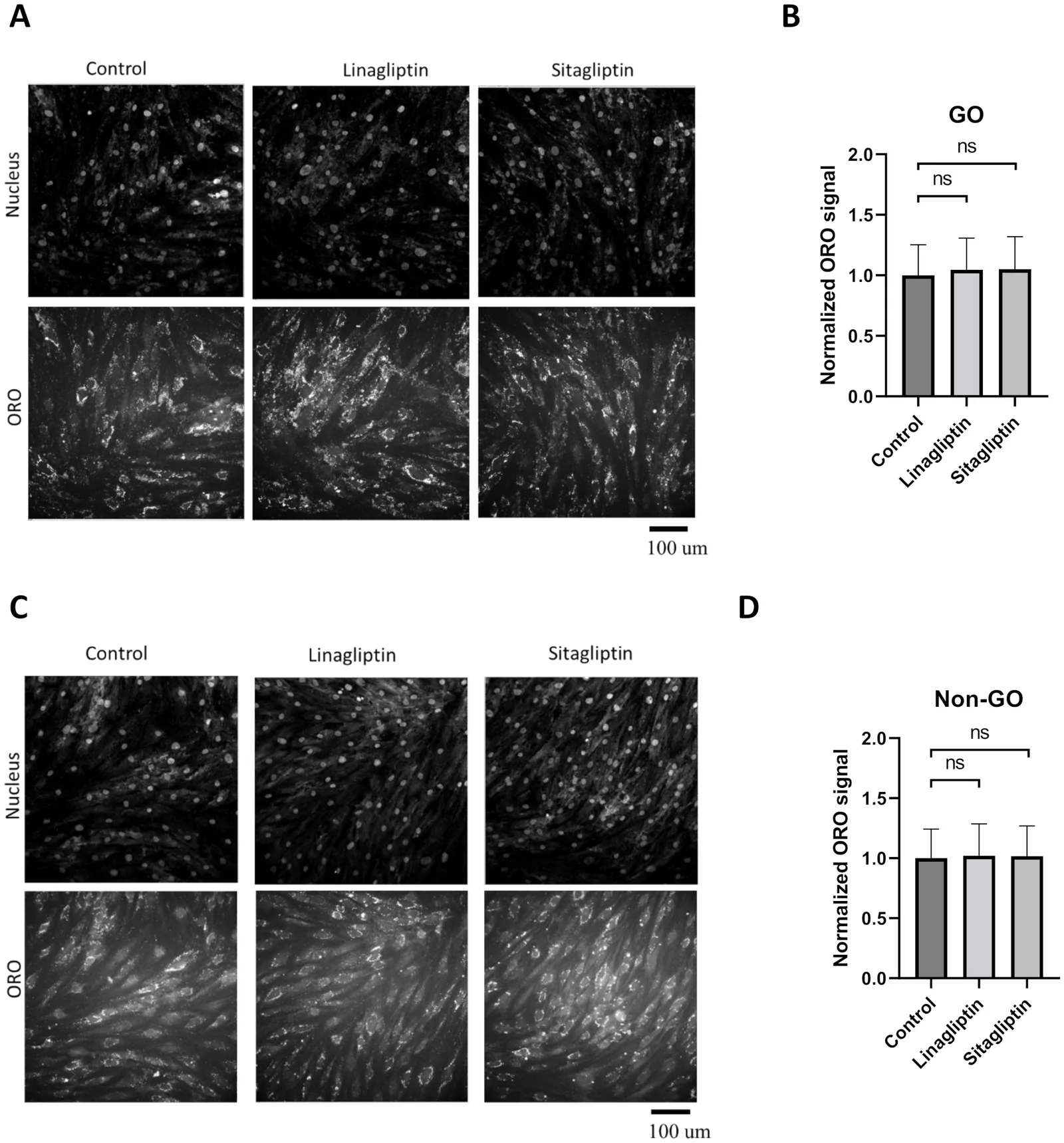
DPP4 inhibitors do not affect Oil Red O (ORO)-assessed adipogenic differentiation in orbital fibroblasts from GO or non-GO subjects. Orbital fibroblasts derived from GO (n = 10 donors) and non-GO (n = 10 donors) subjects were cultured for 12 days in the presence or absence of DPP4 inhibitors, linagliptin (1 µM) or sitagliptin (20 µM). **(A)** Representative ORO staining images of GO orbital fibroblasts. **(B)** Quantification of normalized ORO staining intensity in GO orbital fibroblasts. **(C)** Representative ORO staining images of non-GO orbital fibroblasts. **(D)** Quantification of normalized ORO staining intensity in non-GO orbital fibroblasts. ORO fluorescence intensity was normalized to DAPI-stained nuclei. For each donor, technical replicates were averaged, and values were normalized to the corresponding untreated adipogenic control, which was set to 1. Data are presented as mean ± SD from biological donors. ns, not significant.

Lipid droplets were detected by Oil Red O staining. No significant differences in lipid droplet accumulation were observed after treatment with linagliptin (1 µM) or sitagliptin (20 µM) in either GO (**Figures 4A, B**) or non-GO fibroblasts (**Figures 4C, D**).

### 3.5 DPP4i inhibited IL-1β-induced hyaluronan production in orbital fibroblasts

**Figure 5:**
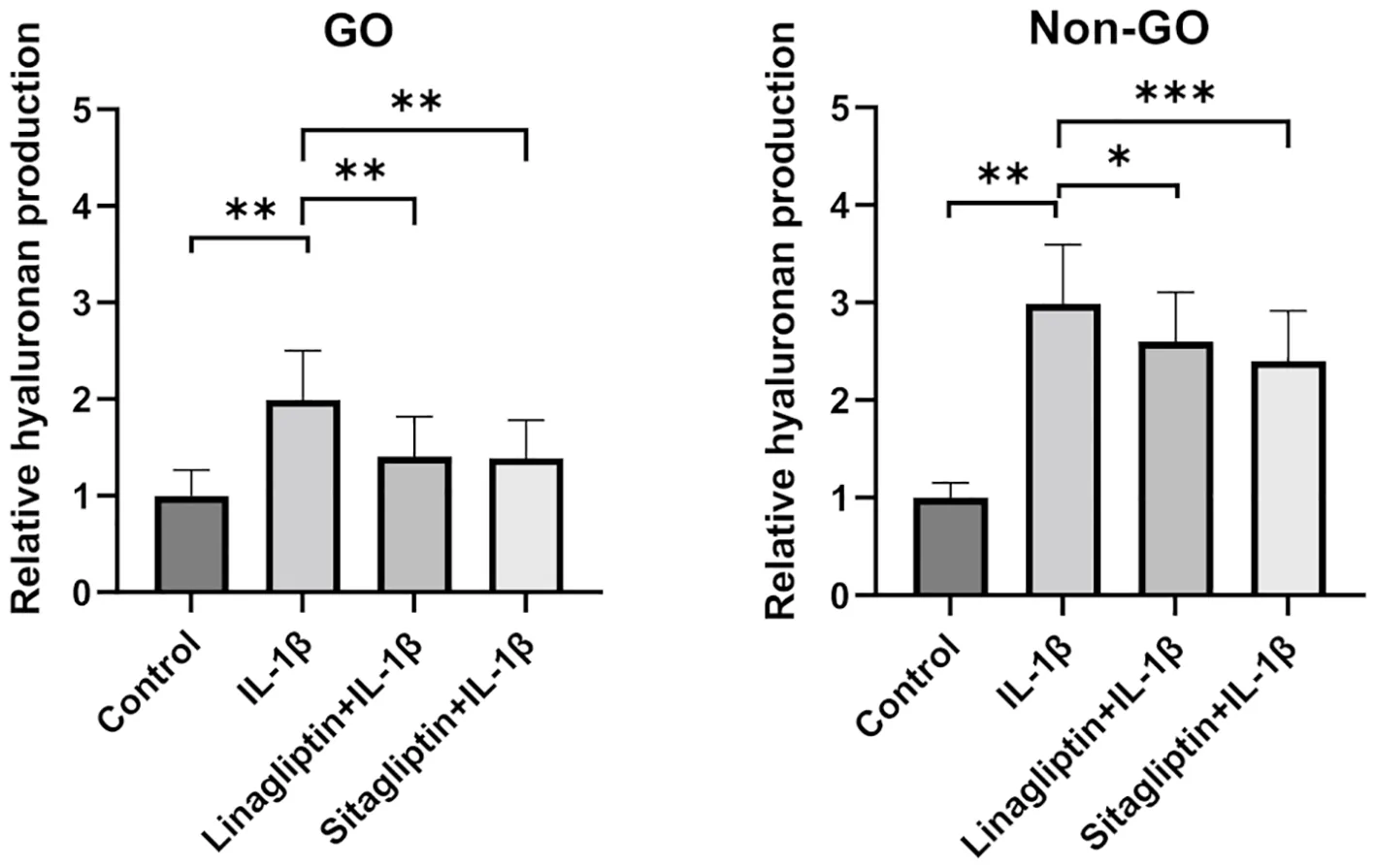
DPP4 inhibitors suppress IL-1β–induced hyaluronan production in orbital fibroblasts. Orbital fibroblasts derived from GO (n = 10 donors) and non-GO (n = 10 donors) were stimulated with interleukin-1β (IL-1β, 10 ng/mL) for 24 hours in the presence or absence of DPP4 inhibitors (linagliptin, 1 µM; sitagliptin, 20 µM). Hyaluronan levels were quantified and normalized to the mean of the corresponding control, which was set to 1. Bars represent mean ± SD from three independent experiments. *P < 0.05; **P < 0.01; ***P < 0.001.

IL-1β stimulation increased hyaluronan production in orbital fibroblasts from both GO and non-GO subjects. Co-treatment with linagliptin or sitagliptin significantly reduced IL-1β-induced hyaluronan production in GO fibroblasts. Similar inhibitory effects were also observed in non-GO fibroblasts. These results indicate that DPP4i attenuated cytokine-induced hyaluronan production in orbital fibroblasts from both groups (**Figure 5**).

## 4 Discussion

DPP4i, widely prescribed for type 2 diabetes, block enzymatic degradation of incretin hormones such as GLP-1, thereby enhancing insulin release, reducing glucagon, and improving glycemic control. To our knowledge, this is the first study to examine the effects of DPP4i on primary orbital fibroblasts derived from patients with GO, particularly their effects on myofibroblastic differentiation and hyaluronan synthesis. Our study demonstrated that DPP4 was detected in orbital fibroblasts from both GO and non-GO subjects. As DPP4 has been implicated in fibroblast activation and matrix remodeling in other tissues (21–24, 27, 29), its expression in orbital fibroblasts suggests a potential relevance to fibroblast-associated processes and provides a rationale for examining the effects of DPP4i in this context. Non-GO fibroblasts showed a clear increase in DPP4 expression after TGF-β stimulation, consistent with DPP4 acting as a TGF-β–responsive marker (24, 30). GO fibroblasts exhibited a modest trend toward DPP4 induction, and the variable magnitude of their responses to TGF-β may reflect differences in the disease-associated microenvironment. These heterogeneous responses may also be related to the intrinsic diversity of orbital fibroblast subpopulations (31, 32).

In this study, we observed that DPP4i linagliptin and sitagliptin significantly reduced α-SMA expression in GO orbital fibroblasts. Previous studies have suggested that DPP4 plays a role in tissue fibrosis and could be a target for preventing fibrosis through inhibition of Smad or ERK phosphorylation (21–24, 26). For example, Soare et al. showed that DPP4 could serve as a marker of activated fibroblasts and a potential target for treating fibrosis in systemic sclerosis (24). Pharmacological inhibition of DPP4 with DPP4i or DPP4 knockdown reduced fibroblast migration, proliferation, contractile protein expression, and collagen release in mouse models of systemic sclerosis (24). Yoshida et al. demonstrated that linagliptin attenuates fibrosis in Tenon fibroblasts following glaucoma surgery (26). Antifibrotic effects of DPP4i have also been reported in the liver, kidney, and heart (21–23, 29, 33, 34). Our results are therefore consistent with evidence that DPP4i modulate fibrotic remodeling across diverse tissues. To explore the potential signaling mechanism underlying the antifibrotic effect of DPP4i, we examined TGF-β/Smad signaling in GO orbital fibroblast lysates. In this subset, TGF-β significantly increased the p-Smad3/Smad3 ratio, and both linagliptin and sitagliptin significantly reduced TGF-β–induced p-Smad3/Smad3 expression. These findings suggest that attenuation of Smad3 phosphorylation may contribute to the inhibitory effect of DPP4i on TGF-β–induced myofibroblastic differentiation in GO orbital fibroblasts. This observation is consistent with previous reports suggesting that DPP4i may attenuate fibrosis in other fibrotic models, partly through modulation of Smad phosphorylation (21, 22, 26). Further studies with larger numbers of biological donors are needed to confirm the involvement of TGF-β/Smad signaling and to explore other potential pathways. Besides, although α-SMA expression is a widely used marker of myofibroblastic differentiation, functional assays, such as collagen gel contraction assays, would further clarify the biological impact of DPP4i on fibroblast contractility.

In contrast, DPP4i did not significantly attenuate TGF-β–driven α-SMA expression in non-GO fibroblasts. Heterogeneity of fibroblast subpopulation composition may be related to the different effects observed between GO and non-GO groups. For example, CD90^+^ (Thy-1^+^) fibroblasts in GO orbital tissue preferentially undergo myofibroblastic differentiation in response to TGF-β (4, 31, 35), and the enrichment of platelet-derived growth factor receptor (PDGFR)α^+^DPP4^+^ fibroblasts in GO orbital tissue is related to profibrotic tissue remodeling (32). Therefore, differences in the relative abundance or responsiveness of these fibroblast subsets may partly explain why DPP4i more clearly suppressed TGF-β–induced α-SMA expression in GO fibroblasts than in non-GO fibroblasts. However, fibroblast subpopulations were not directly characterized in the present study. Future studies using flow cytometry, immunostaining, or single-cell approaches will be needed to clarify whether specific orbital fibroblast subsets are preferentially responsive to DPP4i.

DPP4i did not alter fibroblast proliferation or Oil Red O–assessed adipogenesis in our study. Although DPP4 has been implicated in promoting adipogenesis (36, 37), our data indicate that lipid accumulation is not influenced by linagliptin or sitagliptin. Fibroblast subpopulations may differ in their response to adipogenic stimulation. The lack of responsiveness to DPP4i suggests that orbital fibroblasts may rely primarily on PPARγ and other adipogenesis-specific pathways unrelated to DPP4 (4, 35, 38). However, adipogenic markers such as PPARγ and FABP4 were not examined in this study. therefore, the effect of DPP4i on full adipogenic differentiation should be interpreted cautiously and warrants further investigation. In addition, DAPI nuclei counting does not directly assess DNA synthesis or cell-cycle progression, and BrdU or EdU incorporation assays should be considered in future studies to more precisely evaluate proliferative activity.

In our study, both linagliptin and sitagliptin suppressed IL-1β–induced hyaluronan production. Elevated IL-1β stimulates hyaluronan synthesis and adipogenesis in fibroblasts, making it a relevant *in vitro* model for mimicking the orbital microenvironment (38–41). Dysregulated hyaluronan production represents an upstream driver of tissue fibrosis (42, 43). Hyaluronan also contributes to edema, matrix expansion, and ultimately fibrotic remodeling in Graves’ orbitopathy (44). Our results suggest that DPP4i may represent a potential approach to modulate hyaluronan production in orbital fibroblasts, which could be relevant to orbital tissue remodeling in GO.

Fibroblast heterogeneity may underlie the differing patterns of type 1 GO (fat expansion) and type 2 GO (muscle fibrosis) (4, 38, 39). A PDGFRα^+^DPP4^+^ subset enriched in fibrotic areas, together with CD90^+^ TGF-β–responsive fibroblasts, may contribute to these variations (31, 32). In our study, DPP4i reduced α-SMA and hyaluronan, suggesting they may preferentially modulate fibroblasts with stronger profibrotic or cytokine-driven activity. Taken together, these findings raise the possibility that some fibroblast subsets or GO subtypes may be more responsive to DPP4-targeted therapy. However, further *in vivo* and clinical studies are required. Additionally, reports have linked low DPP4 levels with autoimmune activity (20). Careful monitoring is warranted when DPP4i are used in GO patients. Although DPP4i are already used clinically for glycemic control, the present findings should be interpreted as *in vitro* evidence of fibroblast phenotype modulation rather than evidence of clinical efficacy in GO. Whether DPP4i can influence GO-related tissue remodeling *in vivo* remains to be determined. In addition, the concentrations of DPP4i used in this study represent pharmacologic *in vitro* doses and should not be interpreted as clinically equivalent orbital tissue exposure levels. Because orbital tissue concentrations of linagliptin and sitagliptin after standard clinical dosing remain unknown, the translational relevance of these findings should be interpreted cautiously. Future pharmacokinetic and pharmacodynamic studies are needed to determine whether sufficient DPP4i can be achieved in orbital tissues *in vivo*.

There were several limitations in this study. The sample size was relatively small, which may have limited statistical power and the results should be interpreted cauciously. Various concentrations of DPP4i may be tested to verify the full pharmacodynamic range. Although early-passage fibroblasts were used, phenotypic drift during *in vitro* expansion cannot be completely excluded. Moreover, our *in vitro* model does not replicate the complex cytokine and immune cell interactions of the orbital microenvironment in GO.

In conclusion, DPP4i selectively suppressed markers of myofibroblastic differentiation and hyaluronan production in orbital fibroblasts, without significantly affecting fibroblast proliferation or lipid accumulation. This effect may be advantageous, as it could attenuate tissue fibrosis and expansion without broadly impairing the physiological function of fibroblasts. However, while DPP4i may modulate selected fibroblast phenotypes related to orbital tissue remodeling in GO, its clinical impact remains to be determined. Further mechanistic, pharmacokinetic, *in vivo*, and translational studies are warranted to clarify the clinical relevance of DPP4i in GO.

## Data Availability

The raw data supporting the conclusions of this article will be made available by the authors, without undue reservation.
